# Detection of factors affecting kidney function using machine learning methods

**DOI:** 10.1038/s41598-022-26160-8

**Published:** 2022-12-16

**Authors:** Arezoo Haratian, Zeinab Maleki, Farzaneh Shayegh, Alireza Safaeian

**Affiliations:** 1 Department of Electrical and Computer Engineering, Isfahan University of Technology, Isfahan, 84156-83111 Iran; 2 Department of Community and Family Medicine, Isfahan University of Medical Sciences, Isfahan, Iran

**Keywords:** Machine learning, Health care

## Abstract

Due to the increasing prevalence of chronic kidney disease and its high mortality rate, study of risk factors affecting the progression of the disease is of great importance. Here in this work, we aim to develop a framework for using machine learning methods to identify factors affecting kidney function. To this end classification methods are trained to predict the serum creatinine level based on numerical values of other blood test parameters in one of the three classes representing different ranges of the variable values. Models are trained using the data from blood test results of healthy and patient subjects including 46 different blood test parameters. The best developed models are random forest and LightGBM. Interpretation of the resulting model reveals a direct relationship between vitamin D and blood creatinine level. The detected analogy between these two parameters is reliable, regarding the relatively high predictive accuracy of the random forest model reaching the AUC of 0.90 and the accuracy of 0.74. Moreover, in this paper we develop a Bayesian network to infer the direct relationships between blood test parameters which have consistent results with the classification models. The proposed framework uses an inclusive set of advanced imputation methods to deal with the main challenge of working with electronic health data, missing values. Hence it can be applied to similar clinical studies to investigate and discover the relationships between the factors under study.

## Introduction

Current studies show that people with chronic kidney disease (CKD) have a higher risk for cardiovascular and all-cause mortality^1,2^. Also, some factors like abnormal levels of blood vitamin D and ferritin can increase this risk^3–5^. In view of this matter, a number of studies in recent years have focused on identifying the relationship between vitamin D levels and kidney function indicators using statistical methods^7–9^. Creatinine and estimated glomerular filtration rate (eGFR) are the most commonly used indicators for measuring kidney function in patients. Blood creatinine is a waste substance produced by the body's muscle metabolism. Released in the blood, the only way to excrete it is through the kidneys, so measuring blood creatinine levels can be an indicator for kidney function: higher creatinine levels than a certain range indicates poor kidney function. Glomerular filtration rate (GFR), which is another indicator of kidney function, is the amount of waste products such as creatinine that are excreted through the kidneys in one unit of time. However, because this indicator is difficult to measure, laboratories often estimate its values based on the result of blood creatinine test, referred to as eGFR (i.e., estimated GFR). The estimated value is obtained using a *Cockcroft and Gault* formula ((140 − age) × weight) / (72 × blood creatinine) which is multiplied by 0.85 for females)^6^. High values of eGFR indicate strong kidney function.

The results of studies on the effect of vitamin D level on kidney function indicate many challenges and contradictions in this regard^7^. Some of these studies, emphasizing the negative impact of vitamin D deficiency on kidney disease, confirm the general view and suggest that increasing vitamin D improves kidney function^8^. However, conflicting evidence has been provided by other studies. This evidence reveals a direct relationship between vitamin D levels and blood creatinine levels and raises the possibility that increasing vitamin D levels can negatively affect kidney function.

Agarwal et al. investigated the effect of taking vitamin D tablets (paricalcitol) on creatinine and glomerular filtration rate, by measuring the blood creatinine and urine creatinine levels in a group of kidney patients taking vitamin D tablets over a seven-day period^9^. The results identified a direct relationship between vitamin D and blood creatinine levels, but also indicated that changes in vitamin D levels do not have a significant effect on glomerular filtration rate obtained by measuring clearances of creatinine, urea, and iothalamate. Hence, there is a possibility that increasing the creatinine levels with taking the vitamin D tablets results from increased creatinine generation and is not related to kidney function in excretion of this substance. Moreover, the investigations of Teumer et al. on the relationship between vitamin D metabolites and eGFR indicate an inverse relationship between these two factors^7^. Since the estimated glomerular filtration rate is not measured but derived from serum creatinine level with an inverse relationship, the results of this paper can be interpreted as a confirmation of the direct association of vitamin D with blood creatinine levels.

Most of these studies have used statistical inference methods such as maximum likelihood estimation and Mendelian randomization method to model the data of kidney function and the influential factors under study. This model is capable to discover the relationships between these factors and kidney function.

Meanwhile machine learning methods are approved to be a powerful tool in analysis of electronic health records and blood laboratory tests data^10,11^. Depending on the purpose of the researchers, a wide range of machine learning methods have been applied to these data. For example, classification and regression methods can increase the accuracy of disease diagnosis by predicting disease based on blood tests^12–18^. Furthermore, by using these methods for discovering the relationships and interactions between blood test variables, the value of the blood test parameters that indicate a particular disease can be determined more accurately^19,20^.

Machine learning models due to their interpretability, could be beneficial to aid the medical decisions. More specifically, after developing models to predict disease based on blood parameters as predictors, by interpreting the model and detection of leading predictors in the model, blood test parameters that are risk factors for the disease can be identified^14,21–24^.

In addition, performing laboratory tests is not always as informative as it costs, and repeating some of the tests does not provide additional information about the patient's condition. These tests are referred to as low-value tests. Developing regression and classification models can be used to predict the blood tests, which can identify these low-value tests that are predictable based on previous tests^25–29^.

All of these advantages of machine learning techniques in modeling blood test data reveals the potential power of these methods to be instrumental in determining the association between vitamin D levels and other blood test parameters with creatinine which is the indicator of kidney function. In recent years, successful efforts have been made in similar contexts. Islam et al. used number of different machine learning methods to predict the CKD and identify the risk factors of the disease^30^. The *random forest* was the most accurate model and hemoglobin is detected as the most significant risk factor. The *logistic regression* model has also been confirmed as an accurate model to predict the CKD based on blood factors^31^. Peng et al. predicted acute kidney injury (AKI) using different models where the best models were *linear regression*, *support vector machine (SVM)*, *multilayer perceptron (MLP)* and number of decision tree based ensemble methods such as *lightGBM* and random forest^32^.

On the other hand, investigating the associations between blood test parameters and kidney function using machine learning methods, requires the observed data contain all possible blood test parameters. However, in practice, according to the physician prescription, the recorded observations as the result of different blood tests vary from person to person. This leads to a significant volume of missing values in this data which is the main challenge of analyzing them with machine learning methods^33^.

Here we introduce a new framework to apply the machine learning methods on the blood test data recorded in health records to discover the relationship between blood test parameters and kidney function. The inclusive range of blood test parameters recorded in these data provides a comprehensive report of body health conditions in different organs. Hence, the analysis of such data by capturing the interaction between different mechanisms of the body can provide a stronger basis for discovered information. The rest of the paper is organized as follows. In the method section we introduce the data and the framework used to analyze. Discovered relationships by developing models and validation of these models are discussed in results and discussion section, which is followed by final conclusions.

## Methods

### Data analysis workflow

Interpretation of blood tests by experts is mainly based on the reference range and the exact value of the tests does not have much effect on medical decisions. Therefore, we decided to adopt the same manner in analyzing this data and perform the prediction based on the three categories of lower values (i.e. first class), medium values (i.e. second class), and higher values (i.e. third class) and to use classification methods to predict the placement range of serum creatinine levels.

**Figure 1:**
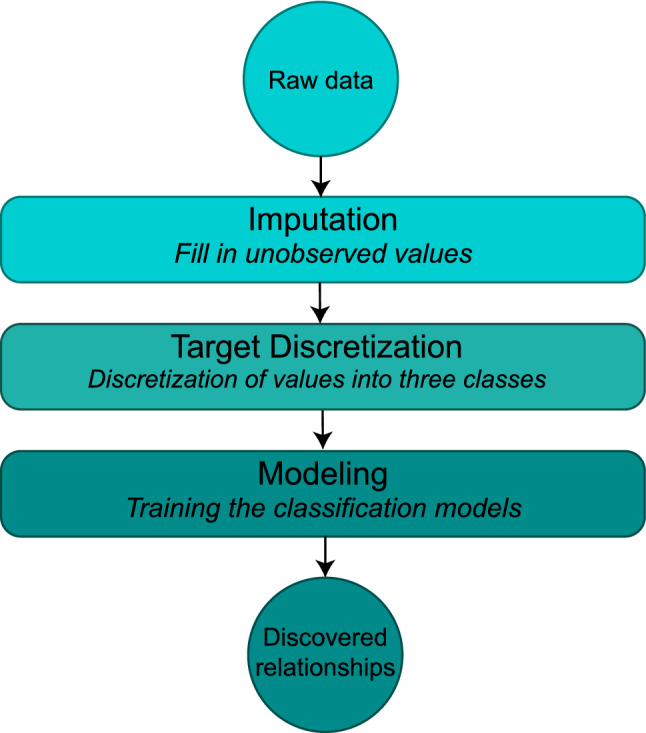
Workflow of the data analysis. Circles show the input and output, rounded rectangles show the base steps, in which the name of step is represented in larger font and the description of the functionality in smaller, italic font.

The modeling framework in this study has a three step procedure, wherein the prediction of the creatinine level for each person is based on the results of other blood test parameters. Figure 1 represents this procedure which takes the raw blood test data as input and discovers the relationships between blood test parameters and creatinine. Training machine learning models for prediction of creatinine level require all values of predictor variables. Thus, in the first step, the values of unrecorded tests for each person (i.e., missing values) must be estimated in an accurate way. There are various methods used to fill the missing values in the data which are referred to as *imputation* methods. However, selecting the efficient method to fill these missing values is of great importance and using simple methods such as replacing the missing values with the average observed values can disrupt the distribution of the variable values. Thus, to estimate the missing values in the first step several advanced machine learning methods are used for imputation.

In the second step *target discretization*, we divide the values of the creatinine level into three categories of the first class, second class, and third class values. We use a data-driven method to obtain these categories in which the cut-off points used to divide the range of values of the creatinine level are selected based on the distribution of these values so that the resulting categories are balanced.

In the third step, *modeling*, several classification methods will be trained to predict the class of creatinine level based on numerical values of the other blood test parameters. Finally, by finding the more important predictor variables in the classification models, which have the greatest impact in detecting the true category of the target variable, the blood test parameters associated with the blood creatinine level are identified. However, the identified associations are reliable in representing the real associations between blood test parameters, only if the model is verified to capture the patterns in the data. The validity of the model can be measured by its accuracy in prediction of the target variable. This is while the accuracy of machine learning methods highly depends on the amount of data used to train the model.

On the other hand, the comprehensiveness of the data used to infer the associations is very important in terms of the absence of bias. More specifically the associations obtained by analyzing the data on the blood tests of people with kidney disease cannot be generalized to the whole population. Hence to obtain general rules, we need to use the blood test data of a group of people with more diverse health conditions. Taking these points into consideration, we have used blood test data recorded in the health records in this study, which includes a significant volume of data recorded from blood tests for both groups of healthy and patient people with a wide range of disease types.

### Data

The data used in this study includes the blood tests of patients referred to Dr. Sharifi Medical Laboratory over a two-year period from 2015 to 2017. Subjects have referred to the lab for periodic checkups or at the doctor's request to check for blood factors, and their informed consent is obtained before tests. All experiments were carried out in accordance with relevant guidelines and regulations. The study was approved by ethics committee of Ministry of Health and Medical Education of Iran.

This data contains 36,309 records, each of which corresponds to one visit to the laboratory. Each record, depending on the reason of the visit, includes some variables of the total 46 blood test parameters able to be measured in the laboratory. The blood test parameters included in the data and percentage of missing values for each parameter are listed in Table 1. As it is clear, the recorded number for each variable in this table represents the percentage of records without any observed value for that variable. For example, the number 41.65 in the table for the hemoglobin variable indicates that 41.65% of the 36,309 records in the data have no value for the hemoglobin variable.

**Table 1:** Name and percentage of missing values for the blood test parameters included in the data.

	Name	Percentage of missing values		Name	Percentage of missing values
1	Hemoglobin	41.65	24	HDL Cholesterol	75.56
2	Hematocrit	41.67	25	T4	77.50
3	Lymphocyte	41.77	26	T3	78.27
4	MCV	41.79	27	Alkaline Phosphatase	78.54
5	Platelets	41.81	28	ESR 1 h	83.83
6	MCH	41.81	29	Ferritin	84.64
7	Neutrophils	41.83	30	Uric Acid	86.52
8	MCHC	41.86	31	Calcium	87.93
9	Monocyte	41.86	32	Phosphorus	90.44
10	WBC	42.05	33	Potassium	91.68
11	RBC	42.09	34	Iron	91.88
12	TSH	55.63	35	pH	92.37
13	FBS	59.22	36	Sodium	92.63
14	Creatinine	62.84	37	Prolactin	92.71
15	Blood Urea Nitrogen	63.30	38	Bilirubin Total	92.98
16	e GFR	65.06	39	Bilirubin Direct	93.06
17	Eosinophil	65.96	40	Hb A1C	93.37
18	Cholesterol	70.27	41	Bilirubin Indirect	93.43
19	Triglycerides	70.36	42	TIBC	94.43
20	SGPT (ALT)	70.41	43	Glucose (2 h p.p.)	96.11
21	Vitamin D (25 OH)	71.15	44	Anti TPO Antibody	96.22
22	SGOT (AST)	71.65	45	Serum Albumin	96.37
23	LDL Cholesterol	75.47	46	Basophil	98.22

To apply the machine learning methods to this data, all the records must have value for the same set of variables. Thus, we use the imputation methods to fill in the missing values of the data.

### Multiple imputation methods

Since strong correlations and interconnections between blood test parameters is one of the most prominent features of this data, taking advantage of these correlations can help to estimate the missing values in the data more accurately. This feature causes the *multiple imputation* methods^34–36^ to be an appropriate option for estimating the missing values in the data. Because multiple imputation methods can take the most advantage of the correlation between variables to make more accurate estimations of missing values. Multiple imputation methods work by iterating the modeling process. Ultimately, this helps to maintain and strengthen the existing patterns in the data.

Multiple imputation methods iterate over a multi-step process to estimate the missing values in the data. In this procedure, first the missing values of all the variables in the data are filled in using a simple method such as mean imputation, and after obtaining the dataset having the initial filled in values, the following steps will be performed for each variable:

1. First the filled in values from the previous step will be removed from the data, and the position of the missing values of the variable becomes empty to be updated.
2. A prediction model will be developed to estimate the values of this variable based on those of other variables in the data. The model is trained using instances having values for this variable.
3. The trained model is used to predict the missing values of the variable.

By repeating this process until satisfaction of a prespecified condition (e.g., reaching the max iterations), the imputed values for the data variables will be converged. Algorithm 1 is the pseudocode of multiple imputation. Here $D$, $P$, and $C$ are the data matrix, prediction model, and stopping condition respectively. $M\left( v \right)$ represents a position of missing values of variable $v$ in the data matrix for $v \in V$ where $V$ is the set of all variables in the data. Obviously, $D\left( {\sim M\left( v \right)} \right)$ is the set of samples in the data matrix for which the value of variable $v$ is not missed.
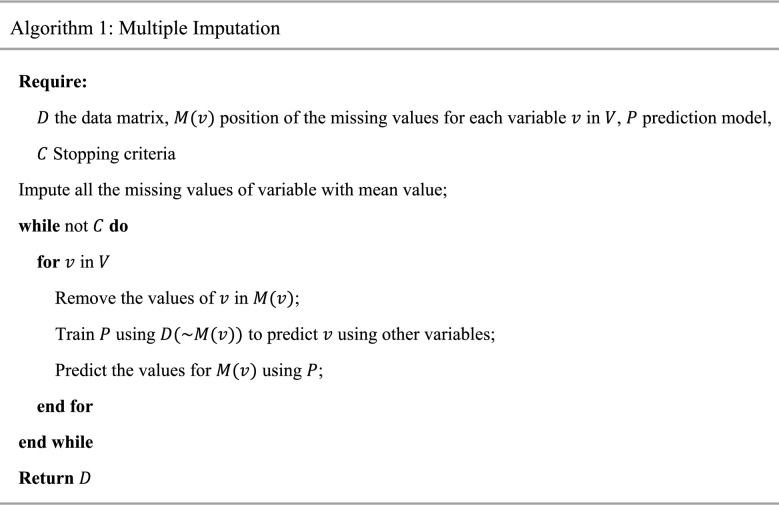


In this paper we used four multiple imputation methods, including *multiple imputation with chained equations*^34^, *multiple imputation with denoising autoencoders*^35^, *missForest*^36^, and *multiple imputation with Bayesian networks*. Each of these methods use a specific model as their core prediction model. The multiple imputation with chained equations uses the *regression* model. The multiple imputation with denoising autoencoders uses *deep neural networks*. The missforest and multiple imputation with Bayesian networks uses *random forest* and *Bayesian network*^37^ respectively, as their core prediction models. In addition to these methods, we also use a common *KNN imputation*^38^ method widely used to fill in the missing values.

As our main goal is to predict creatinine level based on the other blood test parameters as the predictor variables, any leakage of information about the creatinine variable to these predictor variables should be prevented. Thus, we exclude the creatinine from the imputation process and its values are not used to estimate the predictor variables' missing values. After imputing the missing values, the next step for preprocessing the data to apply the classification models is discretizing the target variable.

### Target discretization

The numerical value of blood tests depends on many factors such as physical conditions, drug use and types of nutrition, and can be inaccurate due to laboratory error. Thus, using numerical values of the tests as a target variable in developing the predictive models not only makes it difficult to obtain an acceptable accuracy, but also the patterns identified in models trained based on these values will be unreliable. Therefore, target variable discretization is used to eliminate uncertainty and noise in this data. In the discretization step, the numerical values are mapped to three specific categories of the first, second, and third class.

**Figure 2:**
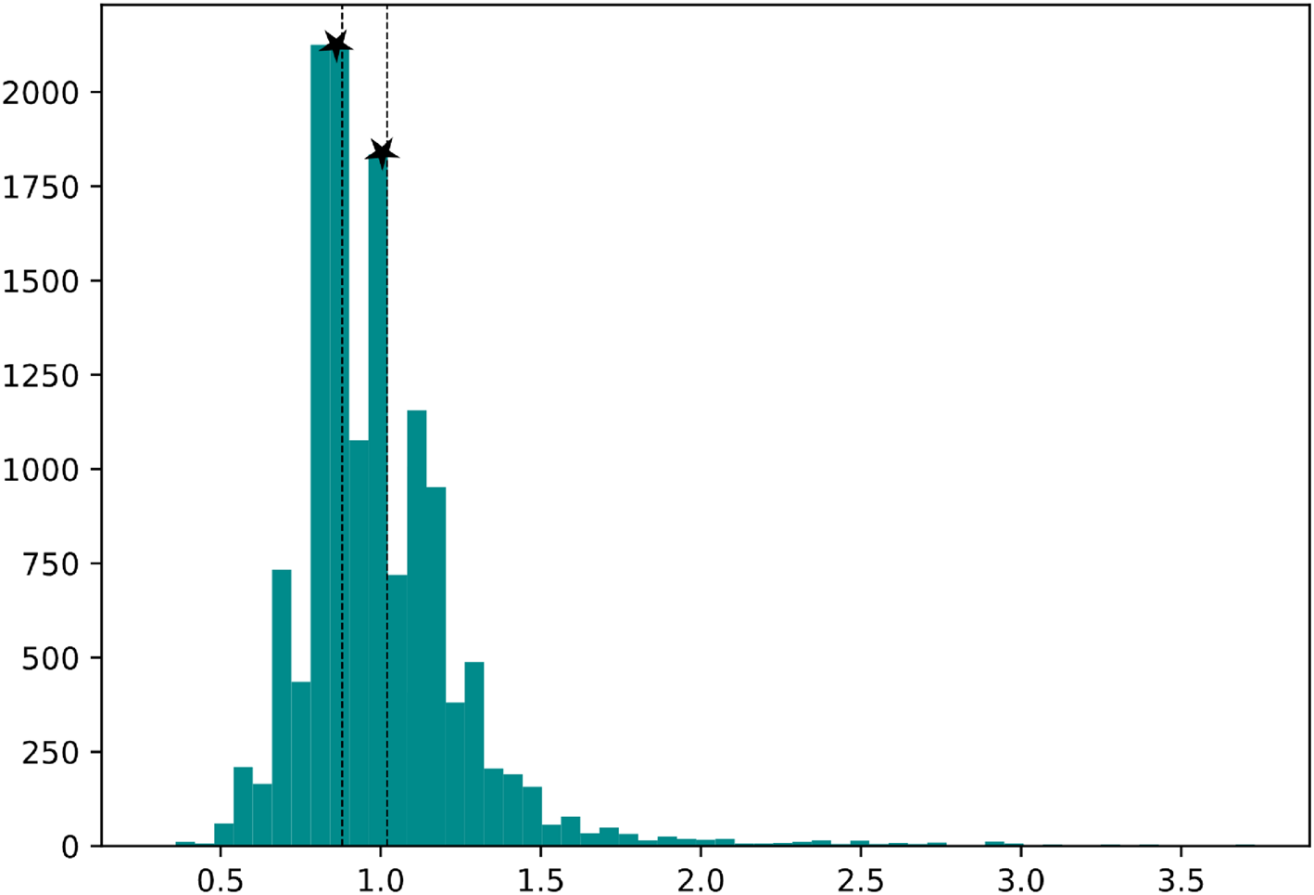
The selected cutting points to discretize the creatinine. The two cutting points at 0.88 and 1.02 are indicated using dashed lines.

The first option that comes to mind for discretization of the blood test parameters (e.g., creatinine) is to use the reference interval to map the test values to the low, medium, and high categories. However, this method can lead to unbalanced categories, which will disrupt the process of developing and evaluating the classification model. So, an alternative method based on data distribution is used for discretization. This method selects the two first peaks of the distribution of the values for the given variable (i.e., two values having the highest frequency in patients' test results for this variable) as cutting points. Selecting the cut points based on this method will result in balanced categories, having almost the same number of samples in each category. Figure 2 represents the cutting points selected to discretize the numerical values of the blood creatinine level in the first, second, and third classes.

### Modeling

#### Classification models

After imputing the predictor variables and categorizing the target variable values, classification methods are trained to predict the creatinine level in the first (i.e., [0.3,0.9)), second (i.e., [0.9,1]), or third (i.e., (1,9.5]) classes. It must be noted that the cut points are rounded to one decimal place.

In this paper seven classification methods were used including support vector machine, logistic regression, random forest, LightGBM, *XGBoost*, *CatBoost* and multilayer perceptron to predict the class of creatinine level. We use the implementation of the Python *scikit-learn* package version 0.24.2 (www.scikit-learn.org), LightGBM package version 3.3.2 (https://lightgbm.readthedocs.io/en/v3.3.2/), XGBoost package version 1.6.2 (https://xgboost.readthedocs.io/en/stable/), and CatBoost package version 1.0.6 (https://catboost.ai/en/docs/concepts/python-quickstart) for these methods.

Support vector machine is a method for classification, wherein the goal is to find one or more hyperplanes in the predictor variables space which is able to separate the samples of different classes^39^. The second method is the logistic regression model, which is defined based on the regression model but combines it with a logistic function to obtain the probability of each class for the sample^40^. The four next models, random forest, lightGBM, XGBoost, and CatBoost methods are the ensemble methods that classifies the samples based on the results of several decision tree models^41–44^. The last model, multilayer perceptron is a multi-layer model with separate parameters for each layer. The output of each layer, which is the input of the next layer, in the simplest case, is obtained from a linear equation based on the inputs. The coefficients of this equation constitute the parameters of this layer, which are learned using an iterative process and based on a specific loss function^45^.

The target variable of these models includes categorized values of creatinine and predictor variables include numerical values of other blood tests listed in Table 1. However, we did not use all the 45 remaining variables in the classification models. Instead, we performed a feature elimination step before applying the classification methods on the data. Since the purpose of developing classification models is to interpret these models and discover patterns and associations between blood test parameters, entering additional information into these models should be avoided as much as possible. Particularly, to identify the parameters that have the greatest impact on the values of creatinine level, it is better to avoid using variables that contain redundant information already included in other variables.

**Figure 3:**
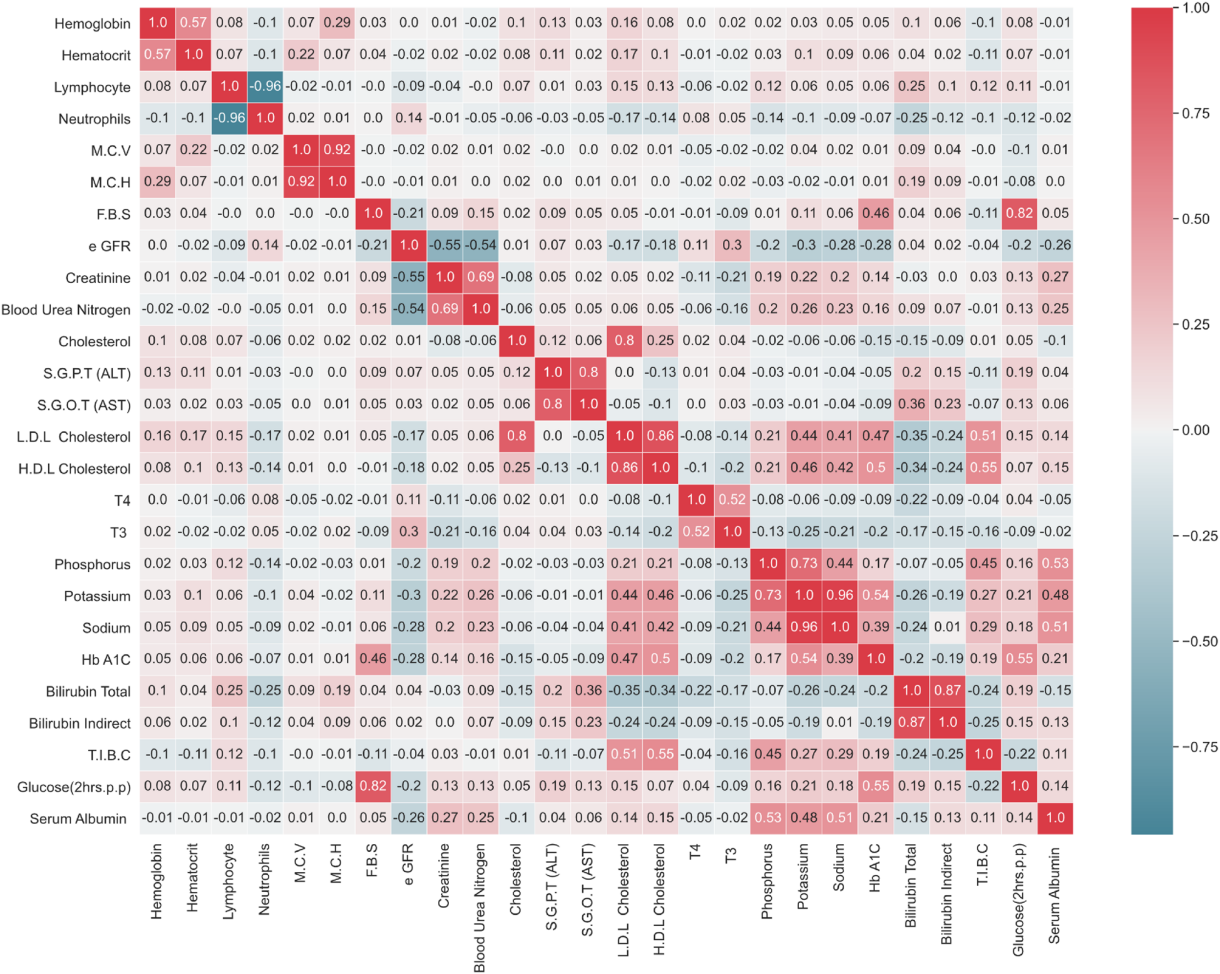
The heatmap plot of correlations between the subset of blood test parameters. The correlation between blood test parameters are used to exclude variables with redundant information from the modeling process.

Figure 3 shows the heatmap plot of correlations between subsets of blood test parameters in the data. As shown in this figure, the correlation between some of the parameters reaches above 0.70. To avoid this additional information entering the classification models, in the feature selection stage, we select only one of the variables from each pair of variables having a correlation more than 50%, to be used in the classification model. As an illustrative example, from the M.C.V and.

M.C.H with correlation of 0.92 only M.C.V are used in the model and M.C.H is excluded from the set of variables. It must be noted that blood urea nitrogen and e GFR that already are known as factors related to the kidney function are excluded in this step.

Before training the classification models, input variables are scaled using the min–max method^46^ to lay in the range between 0 and 1. This step is applied for all the models except random forest, LightGBM, XGBoost, and CatBoost as these models are based on decision trees and do not require variable scaling.

The parameters of the models are selected using grid search method which exhaustively searches over the different combinations of the parameters values to find the values that optimize the classification accuracy. The different parameter values considered for each model are listed in Supplementary Table S1.

The classification models are trained using the preprocessed data and interpreted to reveal the relationships between blood test parameters. However, models like logistic regression can only model the correlational based relationships between variables. Hence to reveal the relationships with probabilistic bases, we decide to use a probabilistic graphical model, namely the Bayesian network.

#### Bayesian network

**Figure 4:**
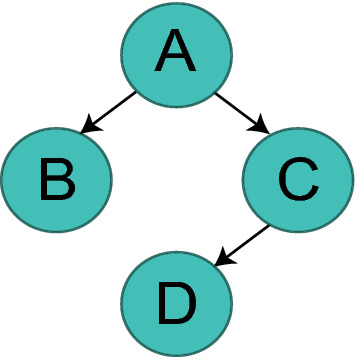
Sample Bayesian network.

The Bayesian network is a graphical model which represents the probability distribution of the parameters using a directed graph. Each node represents a variable, and each edge represents a conditional dependence between the variables. More specifically, if an edge enters from node a to node b, node a is known as the parent of node b. The probability distribution function of the values of each variable in the Bayesian network is dependent on its parents’ values. Moreover, the value of each node given the values of its parents’ nodes is independent of the other variables. Figure 4 represents a sample Bayesian network wherein A is the parent of B and thus probability distribution of values for variable A is dependent on the variable B value.

To verify the relationships found in the classification models, the Bayesian network method was also used to model the relationships between blood test parameters. The reason for using this model is its ability to detect complex and mediated relationships between blood test variables. As our goal is to detect the relationships between blood test parameters and kidney function indicators, inferring Bayesian network structure provides our desired information. The relationships between blood test parameters and kidney function indicators discovered in the classification models can be more reliable, if they are compatible with the relationship patterns in the Bayesian network.

Before extracting the Bayesian network structure from data, we first discretized all the blood test parameters into three classes in a manner similar to that used for the creatinine variable. The resulting discrete values were used for the Bayesian network inference using the implementation of the max–min hill-climbing algorithm by the *bnlearn* package version 4.7 (www.bnlearn.com) in the R programming language.

## Results and discussion

### Imputation accuracy

Regarding the high volume of missing values in the blood test data, the imputation step is of great importance. Indeed, as the classification models are trained based on data which is partly estimated using imputation methods, the accuracy of the estimated values greatly affect the classification models validity and accuracy.

To ensure the accuracy of estimated values to fill the missing values in the data, we evaluate the imputation methods using Mean Squared Error (MSE) measure. To this end we first excluded the 100 observed values for the variables and then compared the imputed values with the original values. As an example, the hemoglobin with 41.65% missing values, includes a value for 21,186 records in the raw data. We remove 100 values of this count, and investigate the accuracy of the imputation method in estimating these 100 values. The MSE measure for each imputation method is listed in Table 2.

**Table 2:** The average and variance of the mean squared error for estimating excluded values of the CBC variables using different imputation methods.

Imputation method	Average MSE	Variance of MSE
Multiple imputation with chained equation	0.0020	2.19e−05
Multiple imputation with denoising auto encoders	0.0063	2.12e−05
MissForest	0.0012	4.88e−06
Multiple imputation with Bayesian networks	0.0009	4.41e−06
KNN imputation	0.0038	5.07e−06

If ${\varvec{x}}_{{\varvec{i}}}$ is the real value of i’th sample and $\overline{\user2{x}}_{{\varvec{i}}}$ is the estimated value for this sample and N is the total number of samples, the MSE measure is calculated using the Eq. (1).

1 $$MSE = \frac{{\mathop \sum \nolimits_{i = 1}^{N} \left( {x_{i} - \overline{x}_{i} } \right)^{2} }}{N}$$

To obtain the average and variance of the MSE for each imputation method, first for each of the CBC variables the MSE of estimated values using this method is calculated. Then the average and variance will be obtained using the resulting error values. The set of variables includes RBC, WBC, Hemoglobin, Hematocrit, Lymphocyte, Neutrophils, Platelets, MCV, MCH, MCHC, Monocyte, and Eosinophil. Note that due to the different range of values of each variable, first the values of the variables are normalized and then the error values are calculated and averaged.

### Accuracy of classification models

By combining five imputation and seven classification methods in the proposed framework, 35 different models were obtained to predict the serum creatinine level in one of the three classes. Standard measures of classification methods were used to evaluate the predictability of the developed models, including accuracy, F1 measure, and ROC curve.

The classification models are trained to predict the blood creatinine level in three classes. However, the ROC curve is basically defined for evaluating the binary classification problems. Thus, we need to use different method to obtain a ROC curve for our classifiers. To this end we first calculated the binary classification ROC curve of each class by considering it as the positive class and the two others as the negative class. The three resulting ROC curves corresponding to each of the three classes were used to derive the overall ROC curve of the model using two different methods, namely micro-averaging and macro-averaging^12^. In the micro-averaging method, first the ROC curve of each class is weighted based on its frequency (i.e., number of instances in the class to total number of instances). Then the overall ROC curve will be obtained by averaging the weighted ROC curves. This is while in the macro-averaging method the unweighted average of the binary ROC curves of classes is considered as the overall ROC curve of the model.

The accuracy simply measures the proportion of samples which are classified in the true class to the total number of samples. But the F1 score, similar to the ROC curve, is originally defined for binary classification problems. Similar to the ROC curve, F1 score can be calculated using micro or macro-averaging methods for multi-class problems, which we have used the macro-averaging method to calculate. To calculate this measure for each class, that class is considered as the positive class and other classes as the negative class. If TP and TN are the number of samples in the positive and negative classes respectively which are classified in the true class, and FP and FN are the number of misclassified samples of the positive and negative classes, F1 score is calculated using the Eq. (2).

2 $$F1 score = \frac{TP}{{TP + \frac{1}{2}\left( {FP + FN} \right)}}$$

**Figure 5:**
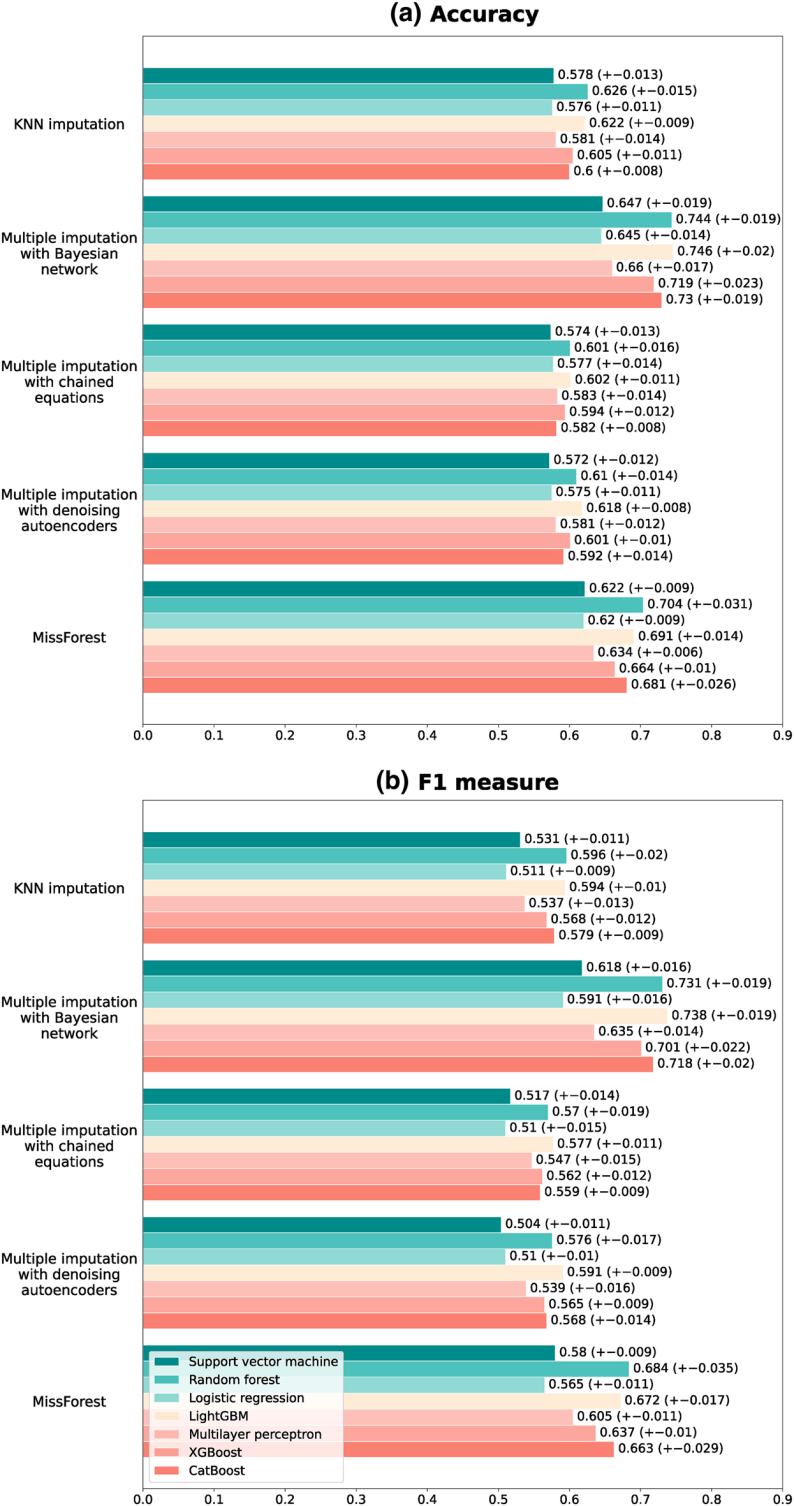
The accuracy and F1 measure of the classification models developed to predict the blood creatinine level. The 95% confidence interval for the criterion is indicated in parentheses.

Figure 5 compares the accuracy and F1 score of different models obtained from combinations of imputation and classification methods. The measures were obtained using a tenfold cross-validation method to ensure the generalizability of the results. Using a cross validation method, data is divided to 10 folds each containing almost 3600 records. Then each time one fold is considered as a testing set and the remaining 9 folds as a training set. The final performance measures for classification models are calculated by averaging the 10 measures obtained for each fold as testing part.

As mentioned before, the accuracy of imputed values in the data can affect the classification models. More specifically inaccurate values for the predictor variables can lead to incorrect prediction for creatinine level. Overall comparison of the different imputation methods shows that the classification models trained using the data obtained from applying Bayesian network imputation method, have the highest predictive accuracy (all have accuracy of more than 0.64). Regarding the relatively high dimension of our data, the superiority of the Bayesian network can be attributed to its capability in modeling high dimensional data^47^.

**Figure 6:**
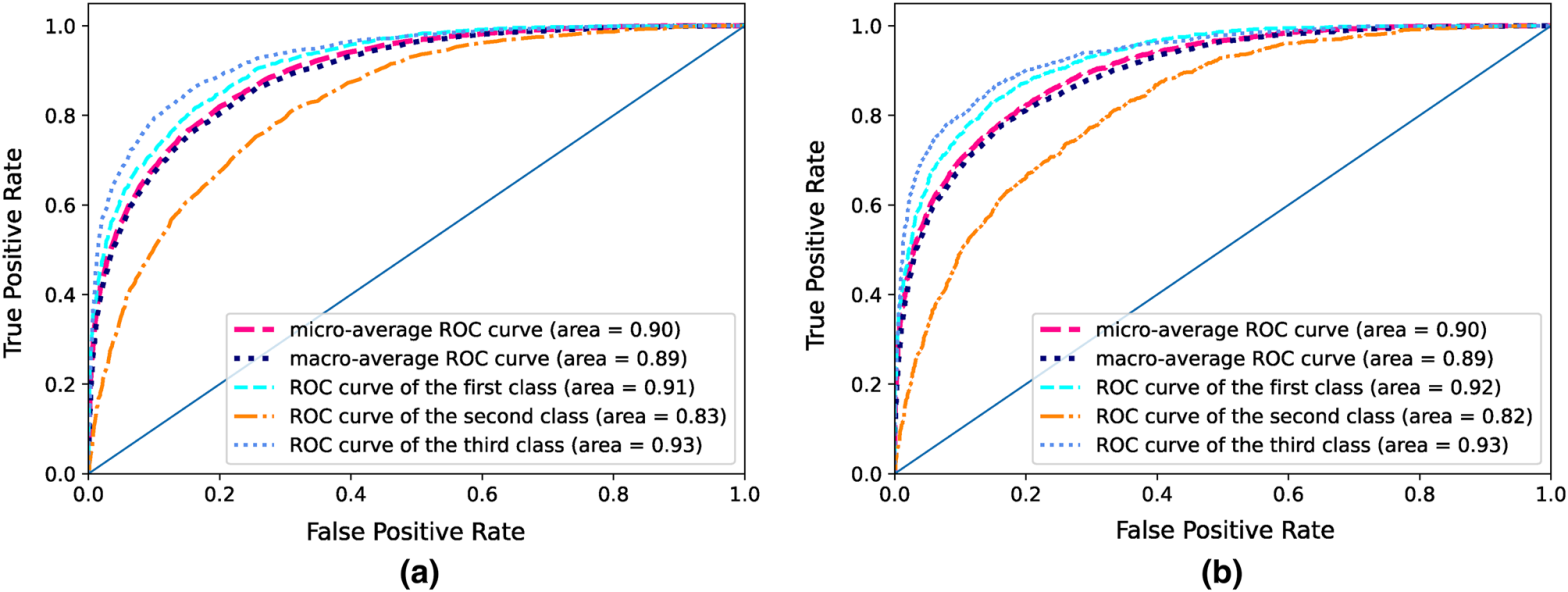
ROC curves of the (**a**) random forest (**b**) LightGBM classification models which is trained to predict the blood creatinine level in three classes. The ROC curves obtained using micro-averaging and macro-averaging methods are plotted along with binary classification ROC curves of each class against other classes.

The comparison of the classification models suggests the random forest and LightGBM as the most accurate methods (always having accuracy more than 0.60). The classification model obtained from a combination of multiple imputation with the Bayesian network and the LightGBM for modeling, has achieved an AUC of 0.90. The same value for AUC is obtained using the combination of this imputation method with random forest model. The ROC curve for these methods is shown in Fig. 6.

**Figure 7:**
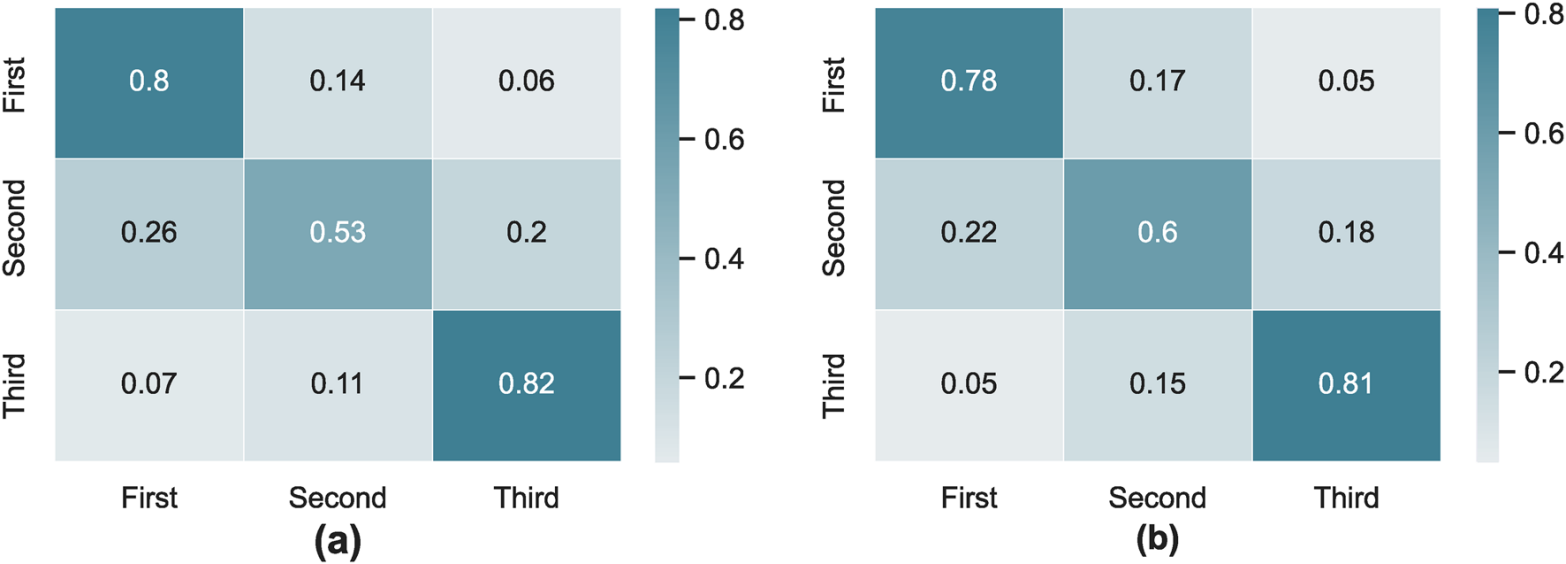
Confusion matrix of the (**a**) random forest (**b**) LightGBM classification models which is trained to predict the blood creatinine level in three classes. Each row indicates the ratio of a samples in a certain class (row label class) that are classified in different classes (column label classes).

The most notable point in Fig. 6 is that comparing the binary ROC curves of three classes, the two curves of the first and third classes cover a larger area of the plot. This may indicate that the results of routine laboratory tests have a significant ability to distinguish samples with abnormal creatinine levels (i.e., above or below normal range) from other samples. The same pattern can be observed in the confusion matrix of these models in Fig. 7.

### Interpretation of classification models

As mentioned before the random forest model is an ensemble model that uses decision trees to classify the samples. In the process of training a decision tree, variables having more capability to differentiate samples of different classes are placed in higher-level nodes.

Based on this, and considering that a random forest model has been developed to classify creatinine level, the selected variables at the decision nodes of the random forest must be examined. Because these variables are the blood test parameters with the most effect on the blood creatinine level.

**Figure 8:**
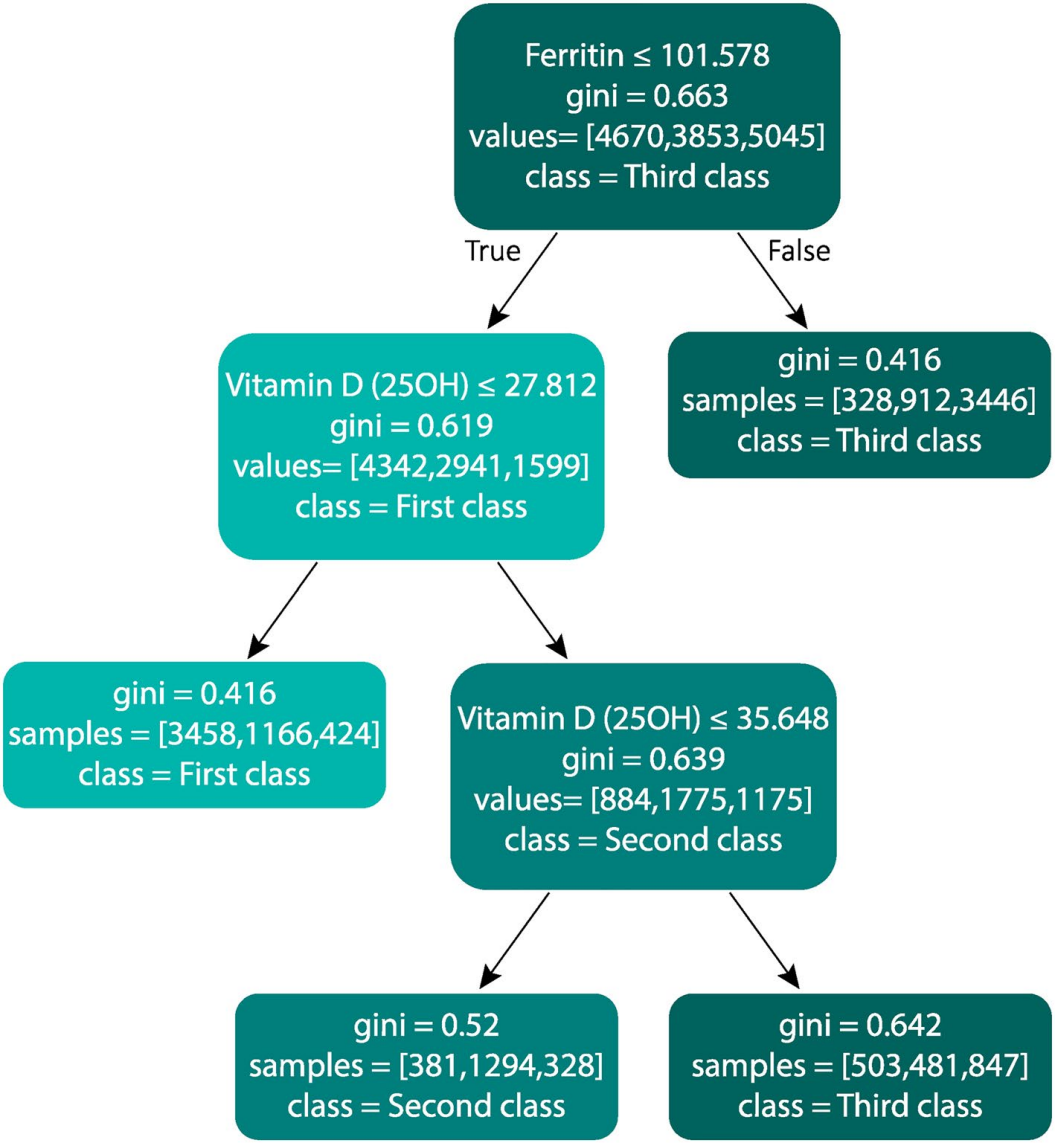
First levels of the random forest decision trees trained to classify the blood creatinine level in three classes.

Figure 8 shows the variables selected at the first levels of random forest trees created to classify the creatinine level. Each node in the tree contains information about the selected variable for splitting, the Gini impurity index, the number of samples belonging to each class, and the dominant class of samples in the node. The number of samples of each class are specified in square brackets and represent the number of samples in the first, second, and third classes, respectively.

Each node of the tree corresponds to a subset of the samples which satisfy the constraints for all nodes above this node (i.e., having greater (less) value than the cutting point for the splitting variable of decision nodes). The Gini impurity index for each node measures the impurity of the samples in the corresponding subset, and less value for this index indicates more consistency of the samples’ classes. This index is used to select a splitting variable and cutting point that will result in subsets with more purity.

The selected variables in the first three levels represented in Fig. 8 are the same in most random forest trees, but the cut points selected for these variables differ for each tree. These slight differences in the cutting points of random forest trees are due to the different samples used in the training of each tree.

As can be seen in Fig. 8, the ferritin and vitamin D levels have been selected as the most influential variables on the creatinine level in the first levels of the tree. Vitamin D levels were selected two times to divide the samples into subsets with higher purity, and each time the class assigned to the right branch of the decision node had higher creatinine levels than the class of the left branch. The first decision node related to vitamin D at the second level of the tree selects the cut point of 27.812 for this variable. Hence, samples for which vitamin D levels were lower than 27.812 are assigned to the first creatinine class and samples with higher levels for vitamin D are allocated to the second creatinine class. Similarly, in the second decision node associated with this variable at the third level of the tree, samples containing values ​​lower than the cut-off point are assigned to the second creatinine class and samples containing values ​​greater than the cut-off point are assigned to the third creatinine class. These observations indicate a direct relationship between the vitamin D and blood creatinine levels. A similar relationship is found between the ferritin and the blood creatinine level at the root node of the tree in Fig. 8. In this node, samples containing values less than 101.578 for the ferritin test are assigned to the first creatinine class, and samples containing values greater than this cut-off point are assigned to the third creatinine class.

The information contained in a tree of Fig. 8 can be summarized in several rules as follows:

- For samples having the ferritin test higher than 101.578, the creatinine test is more likely to be in the third class (i.e. higher values).
- For samples having the ferritin tests lower than 101.578, if the result of the vitamin D level is lower than 27.812, the first class values (i.e. lower values) for the blood creatinine test is the most probable result.
- For samples having the ferritin tests lower than 101.578, if the result of the vitamin D level is between 27.812 and 35.648, the creatinine test is more likely to be in the second class (i.e. medium values).
- For samples having the ferritin tests lower than 101.578, if the result of the vitamin D level is higher than 35.648, the third class values (i.e. higher values) for the blood creatinine test is the most probable result.

### Interpretation of Bayesian models

**Figure 9:**
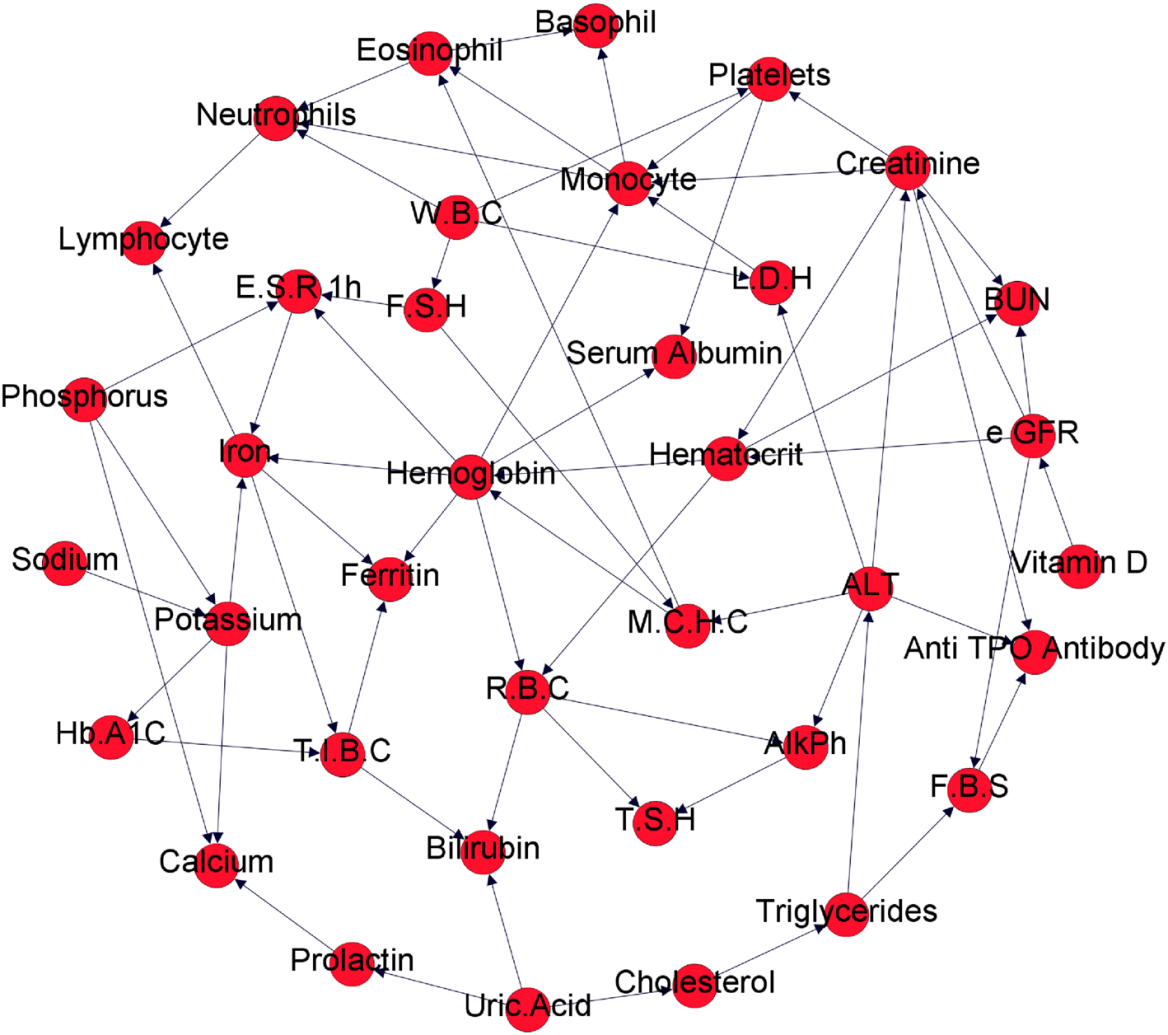
The structure of the blood test parameters inferred from routine blood test data.

In addition to the classification models, the Bayesian network method was used to infer the relationships between the blood test parameters. Figure 9 shows the structure of the Bayesian network of these parameters.

In this network, each node corresponds to a blood test parameter and each edge represents the direct relationship between the two parameters. One of the associations identified in this network is the relationship between the vitamin D level and the eGFR, which is one of the indicators of kidney function.

Table 3 shows the posterior probability of the eGFR indicator given the vitamin D level inferred in the Bayesian network. As shown in this table, the presence of third class eGFR values (i.e. higher values) is most likely when the values of the vitamin D level belong to the first class (i.e. lower values). Moreover, first class values for the eGFR variable are more likely to be present in samples with third vitamin D class. These results indicate an inverse relationship between the vitamin D level and eGFR, and since the eGFR is a derived variable from creatinine with an inverse relationship, this would confirm the direct relationship between vitamin D and creatinine levels detected in the random forest model.

**Table 3:** Posterior probability of the eGFR indicator given the vitamin D level obtained by training the Bayesian network using routine blood test data.

	First class eGFR	Second class eGFR	Third class eGFR
First class Vitamin D (25 OH)	0.24	0.32	0.45
Second class Vitamin D (25 OH)	0.30	0.34	0.30
Third class Vitamin D (25 OH)	0.44	0.32	0.23

### Results comparison

The results of our study are inline with some of the other studies that show the negative effect of ferritin and vitamin D level on kidney function indicators^7,9,48^. However, as a risk factor for kidney disease, previous works does not consider the ferritin or vitamin D level as a potential factor in their models^30–32^. In absence of these factors, Islam et al. detected the hemoglobin as the most significant risk factor for CKD^30^.

On the other hand, using factors such as blood urea nitrogen that are known influential factors on kidney disease is very helpful in development of models which accurately predict these diseases^31^. Nonetheless if the goal is to find the unknown risk factors, it is better to exclude factors like blood urea nitrogen from the model. This allows the model to identify other influencing factors. This can be considered as an advantage of our research compared to previous works.

Moreover, in addition to the common machine learning models, we also used Bayesian network to discover the associations between blood factors. The advantage of this method over other methods and even probabilistic methods used in this literature, such as naive Bayes, is that this method is able to learn probabilistic dependencies between potential risk factors and kidney function indicators.

However, one of the main shortcomings of this research can be considered as the lack of access to more accurate measures of kidney function, such as the GFR factor. Because, as mentioned before, although the increase in blood creatinine level is an important indicator of kidney failure, it may have other reasons such as increased creatinine production in the body.

## Conclusion

Applying machine learning methods to the blood test data resulted in discovering a direct relationship between vitamin D levels and blood creatinine. Our data include the recorded blood tests of all the individuals who have referred to Dr. Sharifi Medical laboratory. This data includes tests for both groups of healthy and patient people which make the inferred relationships generalizable. However, in analyzing this data we were faced with the main challenge of dealing with a high volume of missing values, which was overcome by using multiple imputation methods.

The association between vitamin D levels and blood creatinine is inferred by interpretation of a random forest classification model that is developed to predict the creatinine level. The area under the ROC curve for this model has reached 0.90. The acceptable accuracy of the model guarantees the reliability of the relationships discovered from the model. Moreover, inferring the Bayesian network of blood test parameters from the data has confirmed this relationship.

In conclusion, relying on the detected relationship between vitamin D and blood creatinine levels in machine learning models trained using data including numerous blood test parameters, it can be argued that this relationship exists between these two indicators, independent of some other influential factors on creatinine level that are reflected in the blood test parameters and can form an indirect relationship between vitamin D and blood creatinine.

The discovered relationship between the vitamin D and kidney function indicator, can inform the medical decisions and shed light on the real effect of the interventions like prescribing vitamin D tablets, on the kidney patients health condition.

In addition, there are several other relationships in the Bayesian network of blood test parameters which can be further explored in future works.

## Supplementary Information

Supplementary Information.

## Data Availability

The data used in this study is not publicly available. Access to this data is subject to obtaining permission from Dr. Sharifi Medical Laboratory (drsharifi-lab.com) by contacting the corresponding author.
