# Supplementary material for: Detection of factors affecting kidney function using machine learning methods
Source: Sci Rep. 2022 Dec 16;12:21740. doi: 10.1038/s41598-022-26160-8 (PMC9758148; doi:10.1038/s41598-022-26160-8)
Supplement: Supplementary file 1 — Supplementary Information. [file 41598_2022_26160_MOESM1_ESM.pdf]

# Supplementary materials for “Detection of factors affecting kidney function using machine learning methods”

Arezoo Haratian[1], Zeinab Maleki[1], Farzaneh Shayegh[1], Alireza Safaeian[2]

1. Department of Electrical and Computer Engineering, Isfahan University of Technology, Isfahan 84156-83111, Iran
2. Department of Community and Family Medicine, Isfahan University of Medical Sciences, Isfahan, Iran

Correspondence: Zeinab Maleki
Email: zmaleki@iut.ac.ir

**Table S1.** The parameters and their values used in grid search for each of the different models used in classification of blood creatinine level in the low, medium, and high classes.

| **Model** | **Parameters and values** |
|---|---|
| Support vector machine | kernel: linear, rbf, sigmoid, poly<br>C: 1, 10<br>decision_function_shape: ovr, ovo<br>degree: 2, 3, 4<br>class_weight: None, balanced |
| Logistic regression | solver: newton-cg, lbfgs, liblinear, sag, saga<br>penalty: l1, l2, elasticnet, none<br>dual: True, False<br>C: 0.1, 1, 10<br>class_weight: None, balanced<br>multi_class: ovr, multinomial, auto |
| Random forest | n_estimators: 10, 50, 100<br>criterion: gini, entropy<br>min_samples_split: 2, 3, 4<br>max_features: auto, sqrt, log2, None<br>bootstrap: True, False<br>class_weight: balanced, balanced_subsample, None |
| LightGBM | n_estimators: 10, 50, 100<br>boosting_type: gbdt, dart, goss<br>min_child_samples: 2, 3, 4<br>class_weight: balanced, None |
| XGBoost | n_estimators: 10, 50, 100<br>grow_policy: depthwise, lossguide |

| | |
|---|---|
| | objective: multi:softmax, multi:softprob<br>tree_method: auto, exact, approx, hist |
| CatBoost | iterations: 10, 50, 100<br>loss_function: MultiClass, MultiCrossEntropy, MultiLogloss<br>grow_policy: Depthwise, Lossguide |
| Multilayer perceptron | activation: identity, logistic, tanh, relu<br>solver: lbfgs, sgd, adam<br>learning_rate: constant, invscaling, adaptive<br>hidden_layer_sizes: 1, 2, 3 |
